# Divergent Spatial and Temporal Sensitivities of Forest Biomass to Climate Challenge Space‐For‐Time Assumptions and Models

**DOI:** 10.1111/ele.70466

**Published:** 2026-10-20

**Authors:** Linqing Yang, William R. L. Anderegg

**Affiliations:** ^1^ Wilkes Center for Climate Science and Policy University of Utah Salt Lake City Utah USA; ^2^ School of Biological Sciences University of Utah Salt Lake City Utah USA

**Keywords:** carbon cycling, climate change, earth system models, remote‐sensing, space‐for‐time substitutions

## Abstract

Understanding and projecting forest carbon responses to climate change remains challenging. The widely used space‐for‐time substitution (SFTS) approach assumes that ecological responses along spatial climate gradients can substitute for temporal relationships, yet agreement among spatial, temporal, and mechanistic approaches remains largely unknown. Using global satellite‐derived aboveground biomass (AGB) data, we quantified forest AGB sensitivities to spatial versus temporal climate variability, projected climate‐driven AGB changes under future emission scenarios, and compared results with Earth system models (ESMs). Spatial climate gradients explain substantially more AGB variation than temporal anomalies, with strong divergence across regions and forest ages. ESMs better reproduce spatial sensitivities than temporal ones. Temporal extrapolation generally projects future AGB gains, whereas spatial extrapolation often indicates AGB declines. These contrasts demonstrate that spatial, temporal, and model‐based approaches capture distinct facets of forest–climate coupling, and integrating them can better constrain uncertainty in carbon–climate feedbacks and improve future ecosystem projections.

## Graphical abstract

Spatial and temporal climate–biomass relationships diverge globally: spatial gradients explain more variation and yield much stronger sensitivities than interannual temporal variability. This mismatch propagates into future projections, with spatial relationships indicating forest biomass losses while temporal extrapolations and Earth system models project little change or gains.

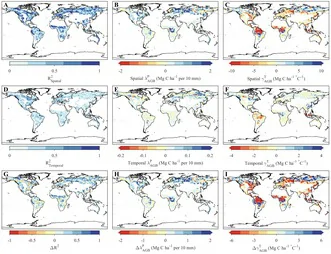

## 1 Introduction

Understanding how climate influences terrestrial vegetation is important for projecting ecosystem responses to climate change and improving Earth system models (ESMs) that simulate the global carbon cycle (Bonan 2008; Friedlingstein et al. 2023). However, existing models of forest carbon cycling have large uncertainties that lead to divergence in forecasts of how climate change will affect the future terrestrial carbon cycle (Piao et al. 2013; Knutti et al. 2014; Arora et al. 2020). Therefore, quantifying the sensitivity of forest dynamics and biomass to climate change is essential for constraining future projections of the terrestrial carbon cycle, informing forest management and conservation, and improving estimates of climate–carbon feedbacks (Bonan 2008; Pan et al. 2011).

Two major empirical approaches that leverage climatic variation are frequently employed to project climate change impacts: temporal sensitivity extrapolation and space‐for‐time substitution (SFTS). The temporal sensitivity extrapolation method assumes that historical climate–vegetation relationships remain stationary through time and can therefore be extrapolated to the future. By contrast, the widely used SFTS method assumes that spatial gradients in vegetation properties along climatic gradients can be used to infer long‐term temporal responses to climate change (Pickett 1989; Lovell et al. 2023). This assumption underpins a broad range of ecological applications, including species distribution models (Blois et al. 2013; Ni and Vellend 2021; Kharouba and Williams 2024), tree growth models (Charney et al. 2016; Babst et al. 2019; Klesse et al. 2020; Wu et al. 2022; Perret et al. 2024), and carbon dynamics models (Estiarte et al. 2016; Coffield et al. 2021; Yang et al. 2022; Uribe et al. 2023; Wu et al. 2023; Roebroek et al. 2024). Both methods have strengths and limitations. Temporal extrapolation directly captures climate impacts but may fail when these relationships become non‐stationary under multiple interacting global change pressures. In addition, constraining climate–vegetation relationships remains challenging due to the limited availability of long‐term observational data across space and time. SFTS captures broader climatic variation and assumes long‐term equilibrium with climate but faces uncertainties related to confounding drivers and historical legacies influencing spatial patterns (Knapp et al. 2017; Evans et al. 2025).

Directly comparing spatial and temporal approaches, together with mechanistic process‐based model simulations that capture plant physiological and demographic responses to climate, can advance our understanding of forest responses to climate change, particularly as ecosystems move into novel climate regimes (Williams et al. 2007). Previous studies, largely focused on tree‐ring growth, have shown that spatial climatic variation models often fail to accurately predict temporal responses to climate change, differing substantially in both magnitude and direction (Klesse et al. 2020; Perret et al. 2024). Perret et al. (2024) and Klesse et al. (2020) leveraged hierarchical mixed‐effects models to capture ring‐width variability in response to spatial and temporal climatic variation for ponderosa pine ( *Pinus ponderosa* ) and Douglas‐fir ( *Pseudotsuga menziesii* ) and found that tree growth derived from spatial climatic variation was often inconsistent with those derived from temporal variability, both in magnitude and even in sign. However, tree‐ring records primarily reflect radial growth of surviving trees and may not fully capture whole‐ecosystem carbon dynamics, including mortality, recruitment, and structural changes, and therefore may not fully represent forest responses to climate variability (Luo et al. 2019). Although permanent sample plot (PSP) studies provide more comprehensive observations of forest structure and dynamics (Luo et al. 2018), they are typically limited to discrete plots, limiting the ability to evaluate the consistency of spatial and temporal climate sensitivities across broad spatial scales.

Despite growing evidence for spatial–temporal divergence in tree growth responses, it remains unclear whether such discrepancies extend to forest biomass, which integrates growth, mortality, recruitment, and disturbance processes. Unlike annual ring‐width increments, aboveground biomass reflects cumulative ecosystem carbon balance and thus provides a more direct constraint on long‐term carbon–climate feedbacks and Earth system model projections (Pan et al. 2011; Anderegg et al. 2020). Furthermore, it remains unknown whether such spatial–temporal mismatches persist across regional to global scales, how they depend on spatial resolution, window extent, and forest age structure, and to what extent empirically derived sensitivities diverge from projections of ESMs. Process‐based land surface models embedded within ESMs simulate coupled carbon, water, and energy fluxes between land and atmosphere, yet continue to exhibit large uncertainties, including overestimation of forest carbon storage and poorly constrained disturbance and CO_2_ fertilization effects (Kolby Smith et al. 2015; Adler et al. 2020; Fisher and Koven 2020; Anderegg et al. 2022; Roebroek et al. 2024). Consequently, confronting empirical spatial and temporal sensitivities with ESM projections is critical for identifying model limitations and improving confidence in future forest carbon projections.

In this study, we used a satellite‐derived global time‐series live woody biomass data set (Xu et al. 2021), the primary component of aboveground biomass (AGB), to quantify and compare the forest AGB response to spatial versus temporal climatic variations. Then, we used these spatial and temporal AGB‐climate relationships to project global forest AGB over the 21st century, comparing these empirical projections to those made by more process‐based ESMs. Specifically, we asked four questions: (1) Are AGB‐climate relationships observed over space similar to those observed over time, and do climate (i.e., temperature and precipitation) exert comparable explanatory power in each method? (2) How does the performance of spatial and temporal relationships differ across spatial resolutions, spatial extents (moving window size), and ecoregions? (3) Do spatial and temporal sensitivities align across forest age classes, and which age groups are most prone to violating the space‐for‐time assumption? (4) How do future projections from spatial versus temporal approaches compare with process‐based ESMs across different time horizons? We hypothesize that (1) spatial models will generally explain more variance in AGB, as spatial gradients integrate long‐term equilibrium responses while temporal models reflect short‐term variability and legacy effects; (2) spatial–temporal sensitivity differences vary across ecoregions, resolutions, extents, and age classes; (3) SFTS will yield more plausible (e.g., consistent with established ecological mechanisms and patterns) projections than temporal extrapolations.

## 2 Data and Methods

### 2.1 Climate Data

We used monthly TerraClimate data for air temperature and precipitation during 2000–2019 at a spatial resolution of 1/24° × 1/24° (Abatzoglou et al. 2018). Climate data during 2015–2100 from 22 Coupled Model Intercomparison Project Phase 6 (CMIP6) ESMs were downloaded from the Earth System Grid Federation (https://esgf‐dev1.llnl.gov/search). We used annual mean temperature and annual total precipitation data under the Shared Socioeconomic Pathways (SSP) 245 and SSP585 scenarios for each year.

### 2.2 Global Forest Aboveground Biomass Data

The Jet Propulsion Laboratory (JPL) living biomass product provides annual estimates (2000–2019) of global living biomass at 0.1° spatial resolution, including both aboveground and belowground biomass. We derived aboveground biomass (AGB) using the root to shoot ratio from Xu et al. (2021). The recent availability of global time‐series AGB datasets has made it possible to quantify and compare spatial and temporal climate sensitivities of forest biomass at the global scale.

Natural disturbances such as fire and insect outbreaks were not explicitly removed, as they represent key mechanisms through which climate impacts are expressed in forest carbon dynamics. We applied a forest mask derived from the global forest management dataset of Lesiv et al. (2022), which provides a widely used and globally consistent classification of forest extent and management status for a reference year (2015). This mask was used to define forested areas and consistently constrain the spatial domain of the analysis across all datasets, restricting analyses to grid cells with > 10% natural forest cover and no or low human impact.

### 2.3 Spatial AGB‐Climate Modelling (Spatial Model)

To investigate the spatial AGB‐climate relationship, we used multiple linear regression for each moving window (Equation 1).

(1) $${\Delta \mathrm{AGB}}_{s}=s0+\gamma_{\mathrm{AGB}}^{T}*{\mathrm{\Delta T}}_{s}+\lambda_{\mathrm{AGB}}^{P}*{\mathrm{\Delta P}}_{s}$$

where ${\text{ΔAGB}}_{s}$, ${\mathrm{\Delta T}}_{s}$, ${\Delta P}_{s}$ is the spatial AGB anomaly, the spatial temperature anomaly, and the spatial precipitation anomaly, respectively. $\gamma_{\mathrm{AGB}}^{T}\;\text{and}\;\lambda_{\mathrm{AGB}}^{P}$ are the regression coefficients, representing spatial sensitivity of AGB to temperature and precipitation, respectively. Regression models were fitted within 5 × 5 pixel moving windows. The spatial anomalies were calculated by removing the mean of the moving windows. Only windows with more than 50% valid grid cells were retained to ensure sufficient sample size. Within each pixel, regressions were performed using all valid pixels, resulting in sample sizes ranging from 13 to 25 pixels per regression. Similar analyses were also performed using 3 × 3‐, 7 × 7‐, 9 × 9‐ pixel moving windows to assess the robustness of spatial and temporal AGB‐climate relationship analysis.

### 2.4 Temporal AGB‐Climate Modelling (Temporal Model)

To investigate the temporal AGB–climate relationship, we applied multiple linear regression models to the annual time series of AGB and climate variables over 2000–2019 at the pixel level (Equation 2).

(2) $${\Delta \mathrm{AGB}}_{t}=t0+\gamma_{\mathrm{AGB}}^{T}*{\mathrm{\Delta T}}_{t}+\lambda_{\mathrm{AGB}}^{P}*{\mathrm{\Delta P}}_{t}$$

where ${\Delta \mathrm{AGB}}_{t}$, ${\mathrm{\Delta T}}_{t}$, ${\mathrm{\Delta P}}_{t}$ is the temporal AGB anomaly, the temporal temperature anomaly, and the temporal precipitation anomaly during the year 2000–2019, respectively. $\gamma_{\mathrm{AGB}}^{T}$ and $\lambda_{\mathrm{AGB}}^{P}$ are the regression coefficients, representing temporal sensitivity of AGB to temperature and precipitation, respectively.

Furthermore, for each of the spatial and temporal analyses, we applied random forest regression (Breiman 2001) using the same input data as the corresponding linear regression models to examine the relationships between pixel‐level AGB and climate variables (temperature and precipitation). Random forest regression has no statistical assumptions (e.g., normality) required of data and is well‐suited for analysing high‐dimensional data. The response and predictor variables were the same as those in the above statistical models. We named the corresponding models as ‘RF‐Temporal’ and ‘RF‐Spatial’ model, respectively. Due to the limited sample size available for each pixel, all valid observations were used for model training. Model performance was internally evaluated using out‐of‐bag (OOB) samples, which are generated during the bootstrap aggregation process and provide an unbiased estimate of prediction error without requiring explicit data partitioning (Breiman 1996). The number of trees was set to 100, as the OOB error stabilized at this value for over 97% of pixels, indicating that this setting ensures adequate predictive performance while maintaining computational efficiency. The JPL AGB and TerraClimate temperature and precipitation data were used for the above‐mentioned models to calculate the regression coefficients.

We acknowledge that the simple linear regression framework of Equations (1) and (2) has challenges and uncertainties related to interpretability, confounding factors, spatial autocorrelation, noise, and saturation effects affecting interannual variation, and the implications of aggregating data across scales. We consider it a reasonable and parsimonious approach to extract climate signals, but complement the analysis with random forest regression to allow non‐linear functional forms and explicitly test the effects of aggregating data to different spatial scales. Given satellite methodological uncertainties and ecological processes captured using different approaches, one might not expect to find the exact same magnitude of climate sensitivities between approaches, but the comparison of the strength, sign, and pattern of sensitivities is nevertheless useful.

### 2.5 Comparison of Explanatory Power and Climate Sensitivities Between Spatial and Temporal Models

The coefficient of determination (*R* ^2^) was used to quantify the explanatory power of both spatial and temporal climate–AGB relationships. We first compared the explanatory power of spatial and temporal models by evaluating differences in *R* ^2^. In addition, we calculated *R* ^2^ for random forest models to assess whether model performance varied among modelling approaches. For each grid cell, we calculated the difference between spatial and temporal model in model explanatory power (Δ*R* ^2^), precipitation sensitivity ($\Delta \lambda_{\mathrm{AGB}}^{P}$), and temperature sensitivity ($\Delta \gamma_{\mathrm{AGB}}^{T}$), where Δ was defined as the spatial estimate minus the corresponding temporal estimate. Positive values indicate stronger spatial sensitivities, whereas negative values indicate stronger temporal sensitivities. To evaluate the validity of the SFTS assumption, we first tested whether Δ differed significantly from zero. Under the SFTS framework, a non‐significant Δ would indicate agreement between spatial and temporal sensitivities, whereas a significant Δ would indicate systematic divergence between the two. To account for spatial autocorrelation among neighbouring grid cells, significance tests were conducted using generalized least squares (GLS) models with an exponential spatial correlation structure defined by geographic coordinates.

We then examined whether the magnitude and variability of Δ depended on methodological and ecological factors. To evaluate how spatial–temporal differences varied across scales, sampling extents, ecosystem types, and forest demographic classes, climate–AGB sensitivities were estimated at multiple spatial resolutions (0.1°, 0.5°, and 1°) and moving‐window sizes (3 × 3, 5 × 5, 7 × 7, 9 × 9, and 11 × 11 grid cells). To assess demographic effects, analyses were further stratified by forest age class (< 50, 50–140, and > 140 years) following Yang et al. (2023). Because age‐specific analyses required sufficient sample sizes within local neighbourhoods, forest age comparisons were conducted only at the 0.1° resolution using an 11 × 11 moving window. Differences among forest age classes were further evaluated using analysis of variance (ANOVA). To assess regional variation, grid cells were also grouped according to the global ecoregion classification of Xu et al. (2021), and the differences of spatial and temporal climate sensitivities were summarized for each ecoregion.

### 2.6 Future Projection of Forest AGB Response to Climate Change

We projected future climate‐driven changes in aboveground biomass (AGB) from 2015 to 2100 relative to the historical period (2000–2014) using climate projections from 22 CMIP6 Earth system models (ESMs) under SSP245 and SSP585 scenarios. Monthly climate data were aggregated into annual variables.

Future AGB responses were estimated using three approaches: (1) direct ESM‐simulated AGB changes, (2) extrapolation based on observational spatial and temporal climate sensitivities, and (3) extrapolation based on ESM‐derived spatial and temporal sensitivities. For the latter two approaches, historical sensitivities were combined with projected climate anomalies to estimate future AGB changes. These extrapolation approaches assume that climate sensitivities under future conditions can be approximated by those observed across present‐day climate gradients.

To evaluate ESM performance, spatial and temporal sensitivities were derived from each ESM over the 2000–2014 period and compared with observation‐based sensitivities using Taylor diagrams. For the direct ESM‐based projection, temporal sensitivities were estimated using a 15‐year moving window centered on each year and combined with ESM‐simulated climate anomalies. Near the boundaries of the study period, the moving window was adjusted to include the nearest 15 available years. To evaluate whether model fidelity in reproducing AGB‐climate sensitivities could serve as an emergent constraint on future AGB projections, we examined the relationship between each ESM's skill and its projected climate‐induced ΔAGB by 2100.

## 3 Results

### 3.1 Degree of Climate‐Vegetation Coupling in Spatial Versus Temporal Approaches

We quantified the spatial‐ and temporal‐ variance in AGB explained by temperature and precipitation both at 0.1° and 1° resolution and evaluated the explanatory power of spatial and temporal models using *R* ^2^. Spatial models explained substantially more variance in AGB (median *R* ^2^ = 0.48 (10th–90th percentile: 0.11–0.81)) than temporal models (median R^2^ = 0.22 (10th–90th percentile:  0.04–0.50)), with the strongest spatial explanatory power in humid tropical and temperate regions (Figure 1A,E). Temporal coupling between interannual climate variability and AGB change was weaker and more spatially heterogeneous. A similar spatial–temporal contrast was observed at 0.1° resolution (median spatial *R* ^2^ = 0.35 (10th–90th percentile: 0.07–0.72); median temporal *R* ^2^ = 0.16 (10th–90th percentile: 0.03–0.42); Figure S1), although overall *R* ^2^ values were lower than at 1*°* (median spatial *R* ^2^ = 0.48 (10th–90th percentile: 0.11–0.81); median temporal *R* ^2^ = 0.22 (10th–90th percentile: 0.04–0.50)) due to greater local variability and reduced signal‐to‐noise ratio at finer spatial scales. Spatial models consistently exhibited higher explanatory power than temporal models (Δ*R* ^2^ median = 0.23, 10th–90th percentile: −0.18–0.63), and the difference was highly significant (*p*_GLS_ < 0.001), confirming a robust divergence between spatial and temporal climate–AGB relationships.

**FIGURE 1:**
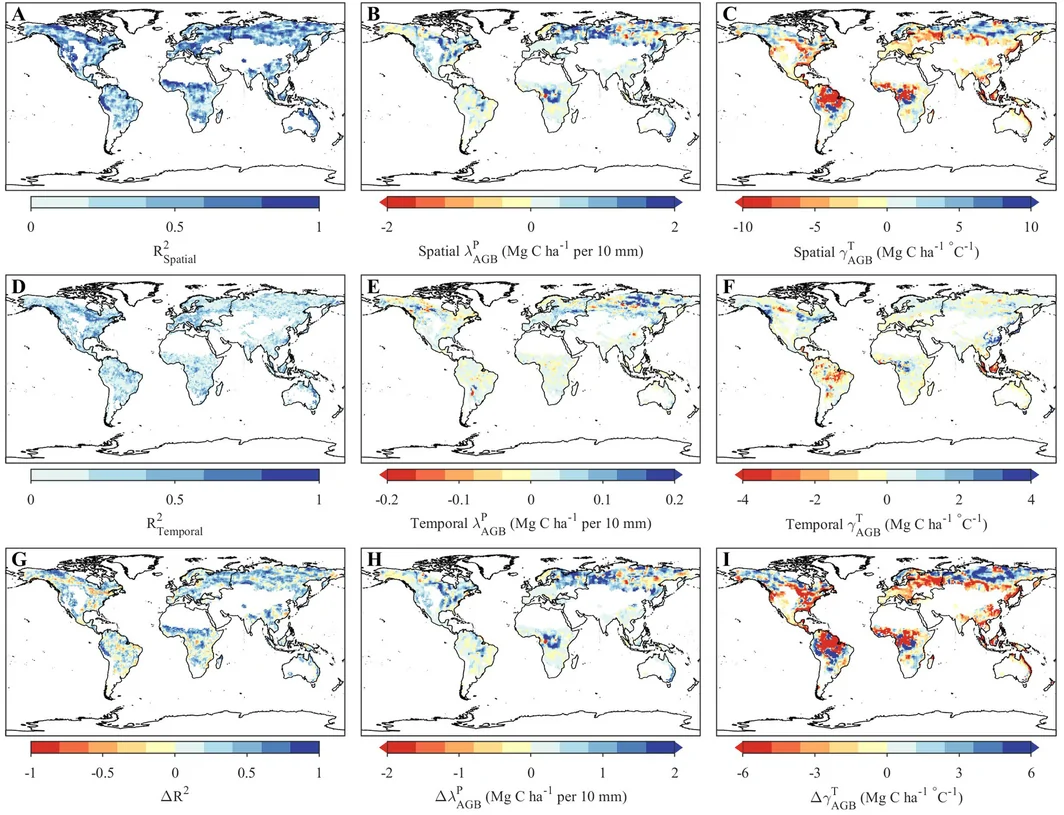
Comparison of climate explanatory power and AGB–climate sensitivities of spatial and temporal model. Panels (A–C) show results derived from spatial models at 1° resolution, including the explanatory power (*R* ^2^; A) and the spatial sensitivities of aboveground biomass (AGB) to precipitation ($\lambda_{\mathrm{AGB}}^{P}$; B) and temperature ($\gamma_{\mathrm{AGB}}^{T}$; C). Panels (D–F) present the corresponding results from temporal models at 1° resolution, including (D) *R* ^2^, (E) temporal $\lambda_{\mathrm{AGB}}^{P}$, and (F)$\;\gamma_{\mathrm{AGB}}^{T}$. Panels (G–I) show the differences between spatial and temporal models, including (G) ΔR^2^, (H) Δ$\lambda_{\mathrm{AGB}}^{P}$, and (I) Δ$\gamma_{\mathrm{AGB}}^{T}$, where Δ denotes the difference between spatial and temporal estimates (spatial−temporal).

Random Forest‐based spatial (RF‐Spatial) and temporal models (RF‐Temporal) exhibited high explanatory power and similar spatial patterns to those of multivariate linear models (Figure S2). RF‐Spatial outperformed RF‐Temporal (area‐weighted mean *R* ^2^: 0.58 (10th–90th percentile: 0.31–0.80) vs. 0.35 (10th–90th percentile: 0.21–0.55)), reinforcing the dominance of long‐term spatial climate constraints over short‐term temporal variability.

### 3.2 AGB Sensitivity to Climatic Variations Differed Across Space Versus Time

Spatial and temporal $\gamma_{\mathrm{AGB}}^{T}$ and $\lambda_{\mathrm{AGB}}^{P}$ also diverged substantially in magnitude and pattern at 1° (Figure 1). Spatial sensitivities were typically an order of magnitude larger than temporal ones. The 10th–90th percentile range of temperature sensitivities ($\gamma_{\mathrm{AGB}}^{T}$) was from −7.77 to 4.99 Mg C ha^−1^ °C^−1^ across space, compared to −0.76 to 0.98 Mg C ha^−1^ °C^−1^ over time. Similarly, the 10th–90th percentile of precipitation sensitivities ($\lambda_{\mathrm{AGB}}^{P}$) ranged from −0.20 to 1.28 Mg C ha^−1^ per 10 mm across space, compared to −0.02 to 0.06 Mg C ha^−1^ per 10 mm over time. Spatial $\lambda_{\mathrm{AGB}}^{P}$ were generally stronger than temporal ones (Δ$\lambda_{\mathrm{AGB}}^{P}$: median = 0.20, 10th–90th percentile: –0.20 to 1.25), whereas $\gamma_{\mathrm{AGB}}^{T}$ were generally stronger in temporal analyses (Δ$\gamma_{\mathrm{AGB}}^{T}$: median = −0.83, 10th–90th percentile: −8.09 to 4.94). Despite substantial variability, all differences were significantly different from zero (*p*_GLS_ < 0.001), confirming a robust divergence between spatial and temporal sensitivities. Spatial patterns also differed: spatial AGB–precipitation slopes were significantly negative (*p* < 0.05) across most forested regions except parts of the northern high latitudes, while temporal slopes were positive in many regions. Spatial and temporal sensitivities were statistically significantly correlated (*p* < 0.05), but showed only weak correlations with each other (|*r*| < 0.15; Figure S3B).

### 3.3 Sensitivity of Spatial and Temporal Slopes to Resolution, Window Size, Forest Age, and Ecoregion

Across all spatial resolutions, moving‐window sizes, forest age classes, and ecoregions, spatial and temporal climate sensitivities differed significantly from one another (all *p*_GLS_ < 0.001; Figure 2), indicating systematic divergence between spatial and temporal climate–AGB relationships. These results challenge the central assumption of space‐for‐time substitution that spatial climate gradients provide unbiased estimates of temporal climate responses. Having established that spatial and temporal sensitivities differ, we next examined whether the magnitude and variability of this divergence depended on methodological choices and ecological factors.

**FIGURE 2:**
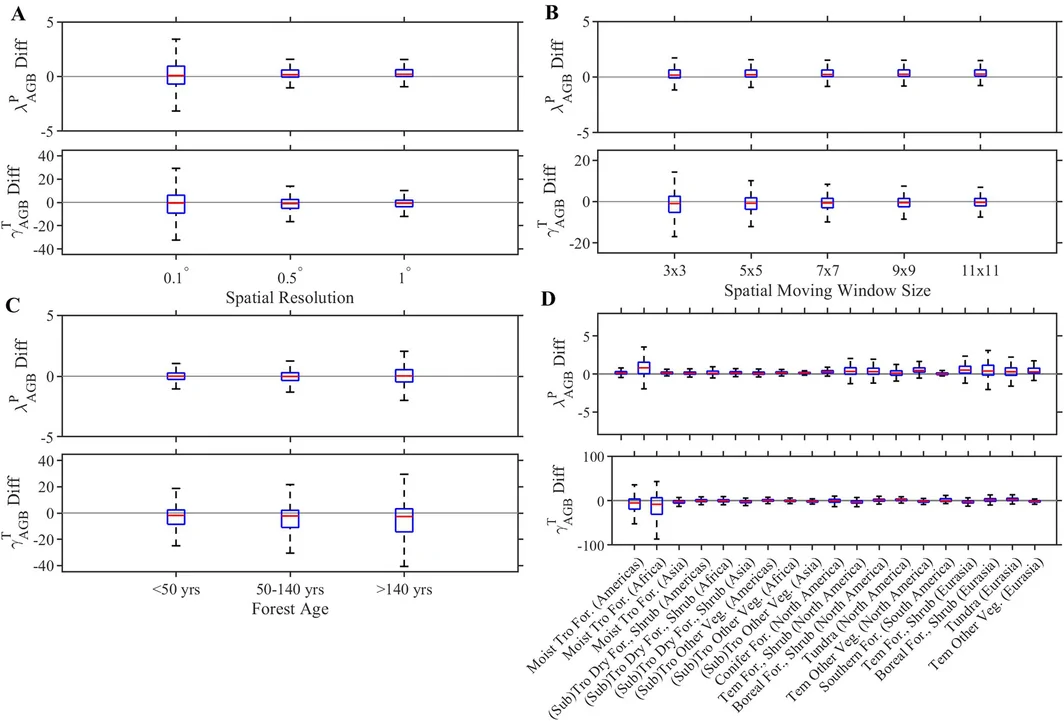
Scale dependence and forest age dependence of spatial vs. temporal AGB–climate relationships. (A) The difference between spatial and temporal sensitivities (slopes) across varying spatial resolutions (0.1°, 0.5°, and 1°). (B) The difference between spatial and temporal sensitivities (slopes) using different moving‐window sizes (3 × 3, 5 × 5, 165 7 × 7, 9 × 9, and 11 × 11 grid cells) at 0.1° resolution. (C) Comparison of spatial vs. temporal AGB–climate sensitivities across forest age classes at 0.1° based on spatial sensitivities estimated using the 11 × 11‐pixel moving window. (D) Spatial–temporal differences in AGB–climate sensitivities across ecoregions, based on spatial sensitivities estimated using an 11 × 11 moving window at 0.1° resolution.

Spatial resolution influenced both the magnitude and variability of spatial–temporal sensitivity differences (Figure 2A). Δ$\gamma_{\mathrm{AGB}}^{T}$ exhibited a clear resolution dependence, with median Δ$\gamma_{\mathrm{AGB}}^{T}$ increasing from −0.58 Mg C ha^−1^ °C^−1^ at 0.1° resolution to −0.47 Mg C ha^−1^ °C^−1^ at 1°, indicating progressively weaker divergence between spatial and temporal temperature sensitivities at coarser spatial resolutions. In addition, finer‐resolution analyses exhibited substantially greater dispersion than coarser‐resolution analyses for both precipitation‐ and temperature‐related differences. Moving‐window size had a modest influence on the average magnitude of Δ$\lambda_{\mathrm{AGB}}^{P}$ but substantially affected the variability of both Δ$\lambda_{\mathrm{AGB}}^{P}$ and Δ$\gamma_{\mathrm{AGB}}^{T}$ (Figure 2B, Figure S4). Smaller windows (3 × 3) produced the widest distributions, whereas larger windows (9 × 9–11 × 11) yielded progressively narrower distributions and more stable estimates. Forest age also affected the spatial–temporal sensitivity differences (Figure 2C; Figure S5). ANOVA detected significant variation among age classes (*p* < 0.05) for both $\lambda_{\mathrm{AGB}}^{P}$ and $\gamma_{\mathrm{AGB}}^{T}$, indicating that stand age exerts a detectable influence on biomass–climate relationships. Although substantial overlap occurred among age classes, middle‐aged forests (50–140 years) exhibited greater variability in both precipitation‐ and temperature‐related sensitivity differences than either young (< 50 years) or old‐growth (> 140 years) forests. Consistent with these patterns, spatial maps revealed that middle‐aged forests displayed the strongest and most spatially heterogeneous climate sensitivities, particularly across tropical and semi‐arid regions (Figure S6). Further subdivision of young forests into 10‐year age intervals revealed that forests younger than 10 years displayed the largest deviations, while little variation was observed among forests aged 10–50 years (Figure S7). At 1°, many grid cells lacked sufficient valid pixels for robust age‐class stratification; therefore, age effects are not included in subsequent analyses. Finally, ecoregion‐specific analyses revealed that the differences were largely consistent across ecoregions, with the exception of tropical moist forests in the Americas (Figure 2D). Most ecoregions exhibited median differences near zero and similar distributional ranges, indicating that the divergence between spatial and temporal sensitivities was not strongly biome‐dependent.

### 3.4 Comparison of Future AGB Projections Across Different Approaches

ESMs differed substantially from observations in both the magnitude and spatial structure of AGB–climate sensitivities (Figures 1 and 3, Figure S8). Across 22 ESMs, temporal sensitivities showed weak correlations with observations (*r* ≈0.1–0.2) and significantly underestimated variance. By contrast, spatial sensitivities were generally better reproduced, with several models achieving correlations > 0.3 and clustering more closely around the observational reference. Nevertheless, large inter‐model spread remained, particularly in the magnitude of sensitivities, underscoring the value of observational benchmarks for constraining future projections.

**FIGURE 3:**
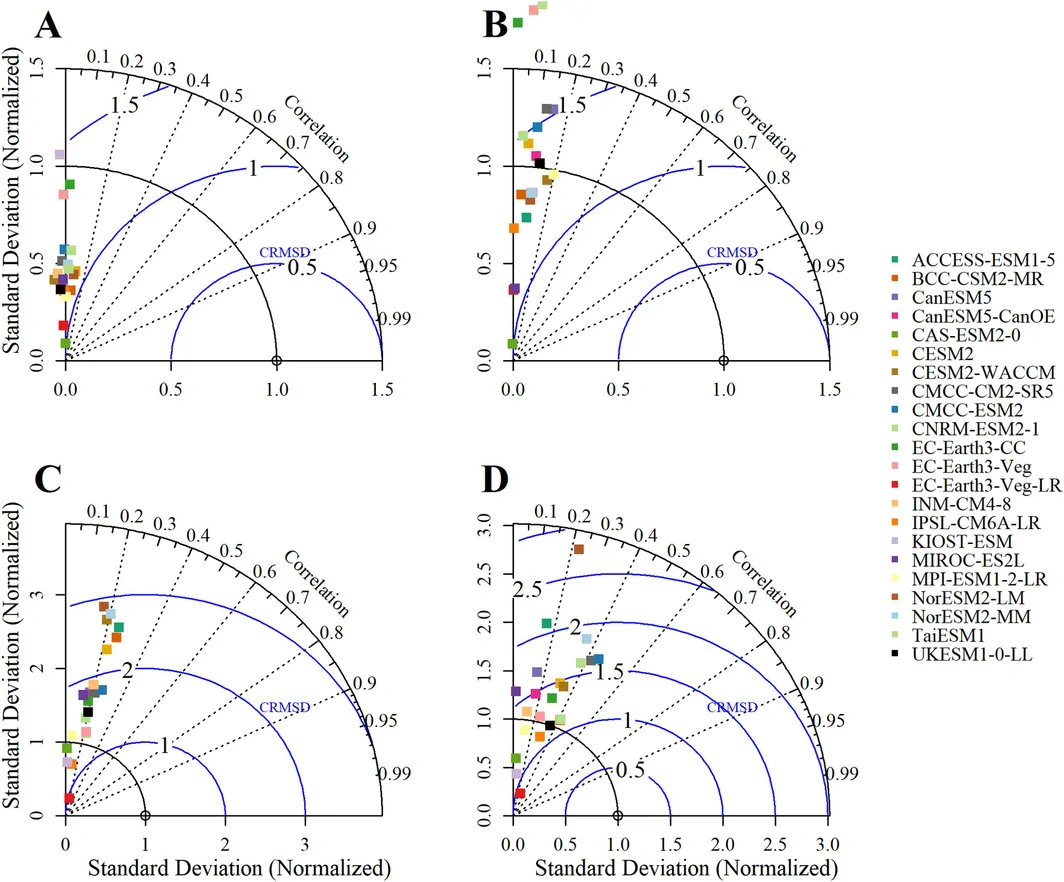
Taylor diagram comparing observed and ESM simulated AGB–climate sensitivities: (A) temporal $\lambda_{\mathrm{AGB}}^{P}$ (B) temporal $\gamma_{\mathrm{AGB}}^{T}$ (C) spatial $\lambda_{\mathrm{AGB}}^{P}$ (D) spatial $\gamma_{\mathrm{AGB}}^{T}$.

Large discrepancies emerged between spatial and temporal extrapolation methods and between empirical and ESM‐based projections (Figure 4). Under both scenarios, observational and ESM temporal sensitivities indicated a slight increase in climate‐driven AGB by 2100, whereas spatial sensitivities projected AGB declines. Under SSP245, observational spatial sensitivities projected relatively modest declines in climate‐driven AGB, largely attributable to temperature‐induced losses partly offset by precipitation‐induced gains. ESM‐based spatial sensitivities simulated a stronger negative trend in AGB change than observations, whereas ESM‐future sensitivities projected a persistent increase in AGB, diverging markedly from both observational and ESM temporal and spatial approaches. Under SSP585, the divergence was more pronounced: ESM simulated larger increases (> 100 PgC by 2100), ESM‐derived spatial sensitivities consistently projected persistent larger AGB declines.

**FIGURE 4:**
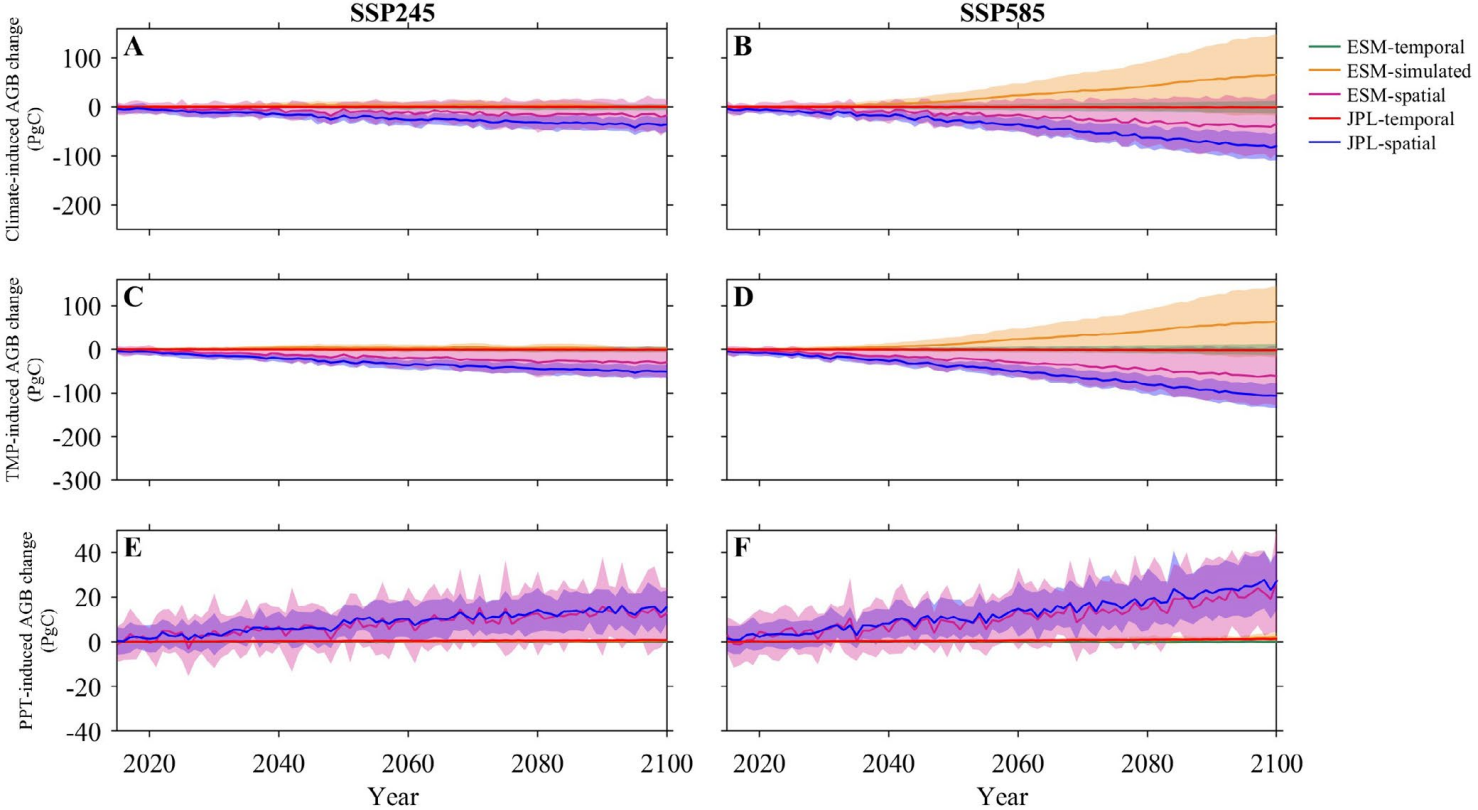
Projected climate‐induced aboveground biomass (AGB) changes relative to the 2015 baseline under SSP245 (left) and SSP585 (right) based on different approaches: (A, B) Total climate‐induced AGB change, (C, D) temperature‐induced AGB change, and (E, F) precipitation‐induced AGB change. Shaded areas indicate the inter‐model spread (±1 SD). Curves represent projection based on ESM temporal historical sensitivities (green), projection based on ESM temporal future sensitivities (orange), projection based on ESM spatial‐historical sensitivities (purple), projection based on observational temporal ‐historical sensitivities (red), and projection based on observational spatial‐historical sensitivities (blue).

Comparisons of climate‐induced AGB changes in year 2100 further emphasized the spatial–temporal contrast (Figure 5, Figures S9–S10). Generally, direct ESM simulations projected widespread increases in AGB, particularly in the northern mid‐high latitudes. In contrast, both observational and ESM spatial sensitivities consistently indicated substantial AGB declines in tropical South America and central Africa—although ESMs significantly overestimated the magnitude of these declines. Temporal extrapolation yielded larger discrepancies: observations revealed localized but pronounced biomass responses, whereas ESMs projected only weak and spatially inconsistent changes. Taken together, these results demonstrate that spatial climate gradients impose stronger and more reliable constraints on biomass distributions than interannual variability, and that ESMs align better with spatial than temporal sensitivities.

**FIGURE 5:**
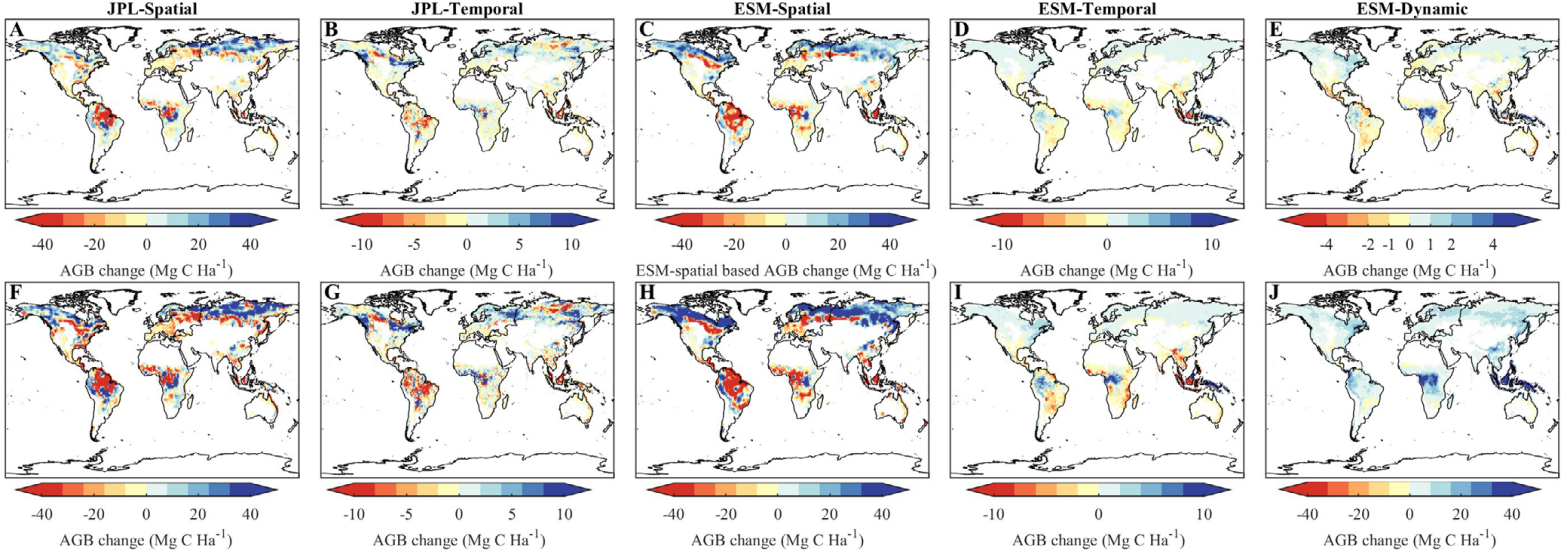
Spatial maps of the global forest climate‐induced AGB change in 2100 relative to the historical period based on observational‐based and ESM‐simulated historical spatial‐ and temporal‐ AGB‐climate relationships, as well as directly simulated ESM AGB changes, at the spatial resolution of 1*°* under (A–E) SSP245 scenario and (F–J) SSP585 scenario, respectively.

Random Forest models based projections showed broadly similar patterns to the statistical models, but substantially smaller magnitude of projected climate‐induced AGB change (Figure S11). Even by 2100, RF‐Spatial projected modest AGB changes relative to the large increases simulated by ESMs. In addition, temperature‐ and precipitation‐induced contributions also differed from those simulated by ESMs.

### 3.5 Emergent Link Between ESM Skill and Projected AGB Change

These systematic differences among models and approaches suggest that variation in model skill may contribute to divergence in projected biomass change. Consistent with this, model skill in reproducing observed AGB–climate relationships was moderately associated with projected future biomass changes (Figure 6). Across scenarios, ESMs that more accurately reproduced observed temperature–AGB relationships generally projected lower temperature‐ and higher precipitation‐induced AGB change. In particular, stronger agreement with the observed spatial or temporal temperature sensitivities was associated with larger declines in temperature‐induced ΔAGB. By contrast, precipitation‐induced ΔAGB exhibited weak or non‐significant relationships with model skill. These findings suggest that fidelity of ESMs to reproduce observed climate–AGB sensitivities provides a potential empirical constraint for improving long‐term carbon‐cycle projections.

**FIGURE 6:**
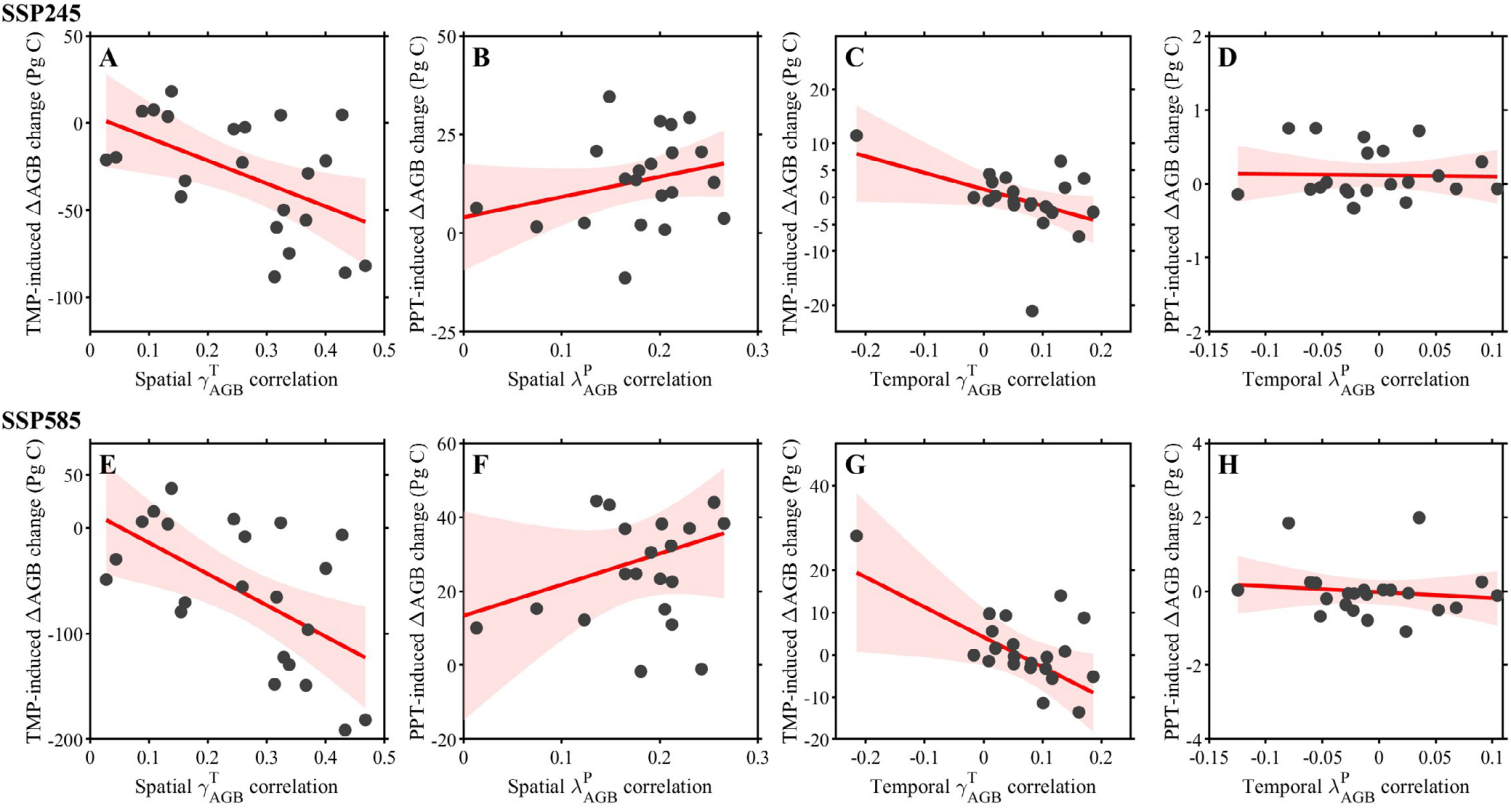
Relationships between ESM skill in reproducing JPL‐based spatial or temporal $\lambda_{\mathrm{AGB}}^{P}$ and $\gamma_{\mathrm{AGB}}^{T}$ and the temperature‐induced (TMP‐induced) or precipitation‐induced (PPT‐induced) AGB change (ΔAGB) in 2100 relative to the historical period under scenarios SSP245 (first row) and SSP585 (second row). Each point represents an Earth System Model (ESM). Each panel shows the regression between ΔAGB driven by temperature (TMP) or precipitation (PPT) and ESM skill of reproducing $\lambda_{\mathrm{AGB}}^{P}$ and $\gamma_{\mathrm{AGB}}^{T}$. Red lines indicate linear fits, and shaded areas represent the 95% confidence intervals.

## 4 Discussion

Our analysis reveals a fundamental divergence between spatial and temporal views of forest carbon–climate coupling, challenging the long‐standing assumption that space‐for‐time substitution (SFTS) can approximate temporal ecosystem responses. Although spatial and temporal approaches are often considered interchangeable under the SFTS framework, our results suggest that they capture distinct ecological processes that operate over different timescales and produce contrasting estimates of climate sensitivity. These differences propagate directly into future AGB projections, leading to opposing conclusions about whether forests will gain or lose carbon under continued warming. Consistent with growing evidence have reported contrasting responses across space versus time for a variety of ecological context including vegetation species distribution (De Lombaerde et al. 2018; Kharouba and Williams 2024), tree growth (Klesse et al. 2020; Wu et al. 2022; Perret et al. 2024), biodiversity (Blois et al. 2013), and phenology (Jochner et al. 2013; Zografou et al. 2020), suggesting that SFTS may not always be a good substitute for temporal response under ongoing climate change.

### 4.1 Divergent Spatial and Temporal Climate Sensitivities

This study highlights a key limitation of the space‐for‐time substitution (SFTS) approach: relationships inferred from spatial gradients do not necessarily translate into temporal dynamics, even when accounting for observational uncertainty. Spatial climate–biomass relationships were substantially stronger and an order of magnitude steeper than temporal relationships. This contrast likely arises from fundamental ecological differences between long‐term equilibrium patterns and short‐term non‐equilibrium dynamics. Spatial gradients integrate persistent biogeographic and ecological adjustments—including species turnover, demographic filtering, soil development, and disturbance legacies—producing quasi‐equilibrium patterns that reflect how forest structure and composition have co‐evolved with mean climatic regimes (Gugger et al. 2010). Temporal sensitivities, in contrast, capture short‐term, non‐equilibrium responses to interannual climate variability and are often obscured by demographic inertia, biological legacies, and stochastic disturbances, resulting in muted or even reversed interannual responses to climate variability.

In addition to these ecological mechanisms, statistical and observational factors further contribute to the apparent divergence. Spatial autocorrelation, as indicated by statistically significant Moran's I tests on the residuals of spatial models across extensive forested regions (Figure S12), inflates the apparent model fit because neighbouring grid cells share similar histories, vegetation structures, and disturbance regimes. Satellite‐derived biomass products are generally more reliable for capturing broad spatial gradients than subtle interannual variability, because of sensor noise, uncertainties in allometric scaling, saturation effects, and data‐fusion limitations. In this study, however, temporal sensitivities are not derived from raw year‐to‐year changes, but from an anomaly‐based regression framework applied across multi‐year time series, which integrates interannual variability and improves the signal‐to‐noise ratio of inferred AGB–climate relationships. We acknowledge that uncertainties in satellite‐derived AGB may still attenuate the magnitude of temporal sensitivities. Importantly, however, such uncertainties are not expected to systematically alter the direction (sign) of the relationships. In contrast, we find that spatial and temporal sensitivities frequently differ not only in magnitude but also in sign. This systematic divergence cannot be explained solely by observational uncertainty, and instead appears to reflect fundamental differences in ecosystem responses across spatial climate gradients versus temporal climate variability.

Together, these mechanisms indicate that spatial and temporal sensitivities represent fundamentally different dimensions of the climate–AGB relationship, underscoring that space‐for‐time substitution cannot be assumed to approximate short‐term biomass responses to ongoing anthropogenic climate change.

### 4.2 Mismatch Between Observations and ESMs Sensitivities

Substantial discrepancies also emerged between observational and ESM‐derived sensitivities. ESMs reproduced spatial sensitivities more faithfully than temporal sensitivities, suggesting that model structures and parameterizations of plant functional types are better tuned to capture long‐term biogeographic gradients than short‐term climate‐driven dynamics. Most ESMs substantially underestimated temporal variance and exhibited weak correlations with observational temporal sensitivities, indicating limited ability to represent interannual growth–mortality dynamics, disturbance‐driven losses, vegetation recovery, and lags in ecosystem carbon allocation (Yang and Anderegg 2023). Improving the spatial representation of climate–AGB relationships can therefore be a central focus for next‐generation terrestrial carbon modelling and emergent constraint frameworks (Fisher et al. 2018; Pugh et al. 2019).

Despite the large model spread, we identified a robust emergent relationship linking ESM skill to projected AGB change: ESMs that better reproduced observed spatial or temporal sensitivities tended to simulate stronger temperature‐driven biomass declines and more positive precipitation effects. This relationship suggests that model fidelity to observed spatial AGB‐climate relationships may serve as a partial emergent constraint on future AGB projections. Improving the model representation of forest climate sensitivities may therefore reduce uncertainty and align projections more closely with empirical constraints.

### 4.3 Comparing Approaches in Projecting AGB Response to Climate Change

Compared with process‐based ESMs, the empirical spatial and temporal approaches did not predict the magnitude of future biomass increases simulated by ESMs, likely due in part to a strong CO_2_ fertilization signal in ESMs. In tropical forests, the negative AGB sensitivity to temperature led to projected AGB losses in the Amazon in both spatial and temporal empirical models, whereas ESMs under high‐emission scenarios simulated continued biomass increases. Uribe et al. (2023) found similar declines in tropical AGB but of a smaller magnitude. Uribe et al. (2023) categorized the climate zones based on the climate space and calculated the equilibrium AGB in each climate zone and found the contraction of humid regions and the AGB loss in the tropical regions AGB over time (when the climate space that one pixel is located changes, the corresponding AGB will change accordingly). In that study, only 19.6% of the entire tropics were projected to move into another climate space, with no projected AGB change in the other regions, which could partially explain the smaller AGB loss projections than in this study. Temperature‐driven biomass losses dominate across methods, whereas precipitation effects remain modest. The stronger $\Delta \gamma_{\mathrm{AGB}}^{T}$ of the spatial model implies that equilibrium responses to chronic warming govern long‐term patterns, while temporal relationships reflect short‐term variability buffered by recovery and acclimation. Existing tree growth studies documented evidence that observed tree growth responses to climate are variable (Astigarraga et al. 2020; Peltier and Ogle 2020) and likely to decrease due to novel climate conditions (Dannenberg et al. 2019; Keen et al. 2022; Liu et al. 2024, 2025). At long forecast time horizons (decades to centuries), climate conditions that have not been historically observed are likely to drive significant forest composition change through colonization and extinction as well as changes in disturbance regimes (Adler et al. 2020). In theory, SFTS should be more robust for projections over long time horizons where the equilibrium assumption may be more reasonable, but this is challenging to validate directly.

### 4.4 Broader Implications for Space‐For‐Time Substitution

Together, our results provide compelling evidence that spatial and temporal sensitivities cannot be treated as interchangeable representations of forest–climate coupling, consistent with recent literature on tree growth. Instead, they reflect fundamentally different ecological processes operating over distinct timescales. The systematic divergence we document challenges the foundational assumption underlying SFTS and suggests that its application to climate change projections should be carefully evaluated, particularly when forecasting short‐ to medium‐term ecosystem responses. Future modelling and empirical efforts should explicitly account for timescale dependence, disturbance–recovery dynamics, and demographic processes to improve the robustness of forest carbon projections.

## 5 Conclusion

By comparing spatial and temporal climate–AGB relationships and their implications for future carbon projections, we demonstrate that space‐for‐time substitution systematically overestimates climate sensitivity relative to short‐term temporal responses and leads to divergent projections of forest biomass change, reflecting long‐term climatic constraints and ecological filtering, whereas temporal relationships capture short‐term, non‐equilibrium dynamics shaped by demographic inertia and disturbance legacies. Current Earth system models reproduce spatial sensitivities more accurately than temporal ones but underestimate interannual variability, contributing to uncertainty in future projections. Together, our findings reveal that spatial and temporal sensitivities represent fundamentally different dimensions of forest–climate coupling, challenging the core assumption of space‐for‐time substitution. Incorporating timescale‐dependent ecological processes, disturbance–recovery dynamics, and improved climate sensitivity constraints into Earth system models is essential for reducing uncertainty and enhancing confidence in future projections of the terrestrial carbon cycle.

## Author Contributions

L.Y. and W.R.L.A. designed the study. L.Y. performed the analyses. L.Y. wrote the first draft and W.R.L.A. provided extensive comments and revisions.

## Funding

This work was supported by the David and Lucille Packard Foundation. National Science Foundation, 1802880, 2003017, 2044937, Alan T. Waterman award IOS‐2325700.

## Conflicts of Interest

The authors declare no conflicts of interest.

## Supporting information


**Figure S1:** The explanatory power (R2) of observational (left column) spatial and (right column) temporal models at the spatial resolution of 0.1°.
**Figure S2:** The explanatory power (R2) of (left column) Random Forest based spatial and (right column) temporal models at the spatial resolution of 1°.
**Figure S3:** Comparison of spatial and temporal sensitivities of forest aboveground biomass (AGB) to temperature and precipitation. Each point represents a 1° grid cell, using all valid paired observations. Red lines indicate ordinary least‐squares regressions. Pearson's *r* and regression equations are shown in each panel.
**Figure S4:** Spatial AGB sensitivity to climate variations at the spatial resolution of 1° based on different spatial moving windows: (first row) 3 × 3 pixels; (second row) 5 × 5 pixels; (third row) 7 × 7 pixels; (forth row) 9 × 9 pixels moving window.
**Figure S5:** Spatial patterns of AGB–climate sensitivities across forest age classes: young (< 50 years), middle‐aged (> 50 years & < 140 years), and old forests (> 140 years). Panels (A–C) show spatial sensitivities of aboveground biomass (AGB) to precipitation (λ_AGB^P^). Panels (D–F) show corresponding sensitivities to temperature (γ_AGB^T^).
**Figure S6:** Comparison of spatial vs. temporal AGB–climate sensitivities for young forests at 10‐year intervals (i.e., < 10, 10–20, 20–30, 30–40, and 40–50 years).
**Figure S7:** Spatial patterns of spatial AGB–climate sensitivities across forest age classes: < 10, 10–20, 20–30, 30–40, and 40–50 years. Left panels show spatial AGB sensitivities to temperature (γ_AGB^T^). Right panels show spatial AGB sensitivities to precipitation (λ_AGB^P^), respectively.
**Figure S8:** ESM simulated spatial (first row) and temporal (second row) AGB sensitivity to precipitation and temperature variations during 2000–2014 based on 22 CMIP6 ESMs at the spatial resolution of 1°.
**Figure S9:** Spatial maps of the global forest temperature‐induced AGB change in 2100 relative to the historical period 2000–2014 based on historical spatial‐ and temporal‐ AGB‐climate relationships and ESM simulated spatial‐ and temporal‐ AGB‐climate relationships at the spatial resolution of 1 degree under (A–C) SSP245 scenario and (D–F) SSP585 scenario, respectively.
**Figure S10:** Spatial maps of the global forest precipitation‐induced AGB change in 2100 relative to the historical period 2000–2014 based on historical spatial‐ and temporal‐ AGB‐climate relationships and ESM simulated spatial‐ and temporal‐ AGB‐climate relationships at the spatial resolution of 1 degree under (A–D) SSP245 scenario and (E–H) SSP585 scenario, respectively.
**Figure S11:** Temperature‐ and precipitation‐induced AGB change during 2015–2100 relative to the relative to the 2015 baseline. Trajectary of (A, B) climate‐induced AGB change (C, D) temperature‐ AGB change and (E, F) precipitation‐induced AGB change predicted by spatial climatic variations (blue), temporal climatic variations (red), and spatial–temporal climatic variations (pink) using random forest regression under (A) SSP245 and (B) SSP585 scenarios with historical randome forest based AGB‐climate trained model. Soild lines and shading indicate mean predictions and standard deviation across 22 different ESMs, respectively. Dash lines indicate the linear regression of spatial‐based AGB change projection.
**Figure S12:** Global distribution of the *p*‐value of Moran's I calculated from residuals of spatial models (5 × 5 window). Blue shading indicates regions with significant spatial autocorrelation (*p*‐value < 0.05), suggesting that residuals in these areas are spatially clustered rather than independent.

## Data Availability

Data and code available via figshare https://doi.org/10.6084/m9.figshare.31374085.
