# Supplementary material for: Divergent Spatial and Temporal Sensitivities of Forest Biomass to Climate Challenge Space‐For‐Time Assumptions and Models
Source: Ecol Lett. 2026 Oct 20;29(10):e70466. doi: 10.1111/ele.70466 (PMC13682585; doi:10.1111/ele.70466)
Supplement: Supplementary file 1 [file ELE-29-0-s001.docx]

**Supplementary material**

**Figure S1:** The explanatory power (R^2^) of observational (left column) spatial and (right column) temporal models at the spatial resolution of 0.1°.

**Figure S2:** The explanatory power (R^2^) of (left column) Random Forest based spatial and (right column) temporal models at the spatial resolution of 1°.

**Figure S3**: Comparison of spatial and temporal sensitivities of forest aboveground biomass (AGB) to temperature and precipitation. Each point represents a 1° grid cell, using all valid paired observations. Red lines indicate ordinary least-squares regressions. Pearson’s *r* and regression equations are shown in each panel.

**Figure S4:** Spatial AGB sensitivity to climate variations at the spatial resolution of 1° based on different spatial moving windows: (first row) 3*3 pixels; (second row) 5*5 pixels; (third row) 7*7 pixels; (forth row) 9*9 pixels moving window.

**Figure S5:** Spatial patterns of AGB–climate sensitivities across forest age classes: young (<50 yrs), middle-aged (>50 yrs & <140 yrs), and old forests (>140 yrs). Panels (A–C) show spatial sensitivities of aboveground biomass (AGB) to precipitation ($\lambda_{\mathrm{AGB}}^{P}$). Panels (D–F) show corresponding sensitivities to temperature ($\gamma_{\mathrm{AGB}}^{T}$).

**Figure S6:** Comparison of spatial vs. temporal AGB–climate sensitivities for young forests at 10-year intervals (i.e., <10, 10-20, 20-30, 30-40, and 40-50 yrs).

**Figure S7:** Spatial patterns of spatial AGB–climate sensitivities across forest age classes: <10 yrs, 10-20 yrs, 20-30 yrs, 30-40 yrs, and 40-50 yrs. Left panels show spatial AGB sensitivities to temperature ($\gamma_{\mathrm{AGB}}^{T}$). Right panels show spatial AGB sensitivities to precipitation ($\lambda_{\mathrm{AGB}}^{P}$), respectively.

**Figure S8:** ESM simulated spatial (first row) and temporal (second row) AGB sensitivity to precipitation and temperature variations during 2000-2014 based on 22 CMIP6 ESMs at the spatial resolution of 1°.

**Figure S9:** Spatial maps of the global forest temperature-induced AGB change in 2100 relative to the historical period 2000-2014 based on historical spatial- and temporal- AGB-climate relationships and ESM simulated spatial- and temporal- AGB-climate relationships at the spatial resolution of 1 degree under (A-C) SSP245 scenario and (D-F) SSP585 scenario, respectively.

**Figure S10:** Spatial maps of the global forest precipitation-induced AGB change in 2100 relative to the historical period 2000-2014 based on historical spatial- and temporal- AGB-climate relationships and ESM simulated spatial- and temporal- AGB-climate relationships at the spatial resolution of 1 degree under (A-D) SSP245 scenario and (E-H) SSP585 scenario, respectively.

**Figure S11**: Temperature- and precipitation-induced AGB change during 2015-2100 relative to the relative to the 2015 baseline. Trajectary of (A-B) climate-induced AGB change (C-D) temperature- AGB change and (E-F) precipitation-induced AGB change predicted by spatial climatic variations (blue), temporal climatic variations (red), and spatial-temporal climatic variations (pink) using random forest regression under (A) SSP245 and (B) SSP585 scenarios with historical randome forest based AGB-climate trained model. Soild lines and shading indicate mean predictions and standard deviation across 22 different ESMs, respectively. Dash lines indicate the linear regression of spatial-based AGB change projection.

**Figure S12**: Global distribution of the *p*-value of Moran’s *I* calculated from residuals of spatial models (5×5 window). Blue shading indicates regions with significant spatial autocorrelation (*p-value* < 0.05), suggesting that residuals in these areas are spatially clustered rather than independent.
